# [^18^F](2*S*,4*R*)-4-Fluoroglutamine as a New Positron Emission Tomography Tracer in Myeloma

**DOI:** 10.3389/fonc.2021.760732

**Published:** 2021-10-12

**Authors:** Silvia Valtorta, Denise Toscani, Martina Chiu, Andrea Sartori, Angela Coliva, Arianna Brevi, Giuseppe Taurino, Matteo Grioni, Livia Ruffini, Federica Vacondio, Franca Zanardi, Matteo Bellone, Rosa Maria Moresco, Ovidio Bussolati, Nicola Giuliani

**Affiliations:** ^1^Department of Medicine and Surgery and Tecnomed Foundation, University of Milan Bicocca, Milano, Italy; ^2^Department of Nuclear Medicine, San Raffaele Scientific Institute, Istituto di Ricovero e Cura a Carattere Scientifico (IRCCS), Milano, Italy; ^3^Department of Medicine and Surgery, University of Parma, Parma, Italy; ^4^Department of Food and Drug, University of Parma, Parma, Italy; ^5^Division of Immunology, Transplantation and Infectious Diseases, San Raffaele Scientific Institute, Istituto di Ricovero e Cura a Carattere Scientifico (IRCCS), Milano, Italy; ^6^Nuclear Medicine, “Azienda Ospedaliero-Universitaria di Parma”, Parma, Italy; ^7^Institute of Bioimaging and Molecular Physiology, National Research Council (IBFM-CNR), Milano, Italy; ^8^Hematology, “Azienda Ospedaliero-Universitaria di Parma”, Parma, Italy

**Keywords:** myeloma, glutamine, metabolism, positron-emission tomography, mouse model, nuclear medicine

## Abstract

The high glycolytic activity of multiple myeloma (MM) cells is the rationale for use of Positron Emission Tomography (PET) with ^18^F-fluorodeoxyglucose ([^18^F]FDG) to detect both bone marrow (BM) and extramedullary disease. However, new tracers are actively searched because [^18^F]FDG-PET has some limitations and there is a portion of MM patients who are negative. Glutamine (Gln) addiction has been recently described as a typical metabolic feature of MM cells. Yet, the possible exploitation of Gln as a PET tracer in MM has never been assessed so far and is investigated in this study in preclinical models. Firstly, we have synthesized enantiopure (2*S*,4*R*)-4-fluoroglutamine (4-FGln) and validated it as a Gln transport analogue in human MM cell lines, comparing its uptake with that of ^3^H-labelled Gln. We then radiosynthesized [^18^F]4-FGln, tested its uptake in two different *in vivo* murine MM models, and checked the effect of Bortezomib, a proteasome inhibitor currently used in the treatment of MM. Both [^18^F]4-FGln and [^18^F]FDG clearly identified the spleen as site of MM cell colonization in C57BL/6 mice, challenged with syngeneic Vk12598 cells and assessed by PET. NOD.SCID mice, subcutaneously injected with human MM JJN3 cells, showed high values of both [^18^F]4-FGln and [^18^F]FDG uptake. Bortezomib significantly reduced the uptake of both radiopharmaceuticals in comparison with vehicle at post treatment PET. However, a reduction of glutaminolytic, but not of glycolytic, tumor volume was evident in mice showing the highest response to Bortezomib. Our data indicate that [^18^F](2*S*,4*R*)-4-FGln is a new PET tracer in preclinical MM models, yielding a rationale to design studies in MM patients.

## Introduction

Multiple myeloma (MM) is a hematological disease characterized by the accumulation of malignant plasma cells (PC) into and, more rarely, outside the bone marrow (BM) (1). In the last years, 2-deoxy-2-[^18^F]fluoro-D-glucose positron emission tomography/computed tomography ([^18^F]FDG PET/CT) in MM has attained significant relevance, and it is considered the cornerstone of MM imaging at the initial diagnosis as well as in staging, prognostic evaluation, and monitoring response to therapy (2). Thus, [^18^F]FDG PET/CT is currently used to assess active bone lesions and extramedullary localizations in MM patients (3). However, [^18^F]FDG uptake yields both false positive and false negative lesions, and only 60–70% of patients with active MM are positive for [^18^F]FDG PET (4, 5). These data support the need for additional imaging methods to assess skeletal involvement and monitoring the effect of treatment. To this purpose, several other PET tracers, such as choline and methionine, have been proposed (6, 7).

[^18^F](2*S*,4*R*)-4-fluoroglutamine ([^18^F]4-FGln) has been recently tested in different types of glutamine (Gln)-dependent tumors (8–10). MM is a Gln-addicted cancer that strictly relies on extracellular Gln uptake, and the use of Gln for anaplerosis has been also confirmed in patients (11, 12). However, [^18^F]4-FGln as a PET tracer in MM has not been investigated yet.

Previous studies showed that [^18^F]4-FGln is taken up by Gln transporters, including ASCT2, in solid tumors (13). Since we have already demonstrated that MM cells have increased expression of several Gln transporters and mainly depend on ASCT2 for Gln transport (11), we hypothesized that [^18^F]4-FGln could be exploited to image MM. In the present study, we firstly documented that 4-FGln and Gln share the same transporters in human MM cell lines. Then, we investigated the sensitivity of [^18^F]4-FGln in comparison to [^18^F]FDG for the detection of MM cells using either a syngeneic murine model or a xenograft model of MM. Lastly, we explored the use of [^18^F]4-FGln to monitor the effects of Bortezomib against MM.

## Materials and Methods

### Chemical Synthesis and Characterization

The synthesis of the four stereoisomers of 4-FGln and their ^18^F-labeled counterparts, together with data on the uptake of these compounds in 9L and SF188-Bcl-xL tumor cells (Gln-addicted glioblastoma-derived tumor cells), has been previously reported (14). In that work, the radiolabeled (2*S*,4*R*)-configured 4-fluoroglutamine [^18^F]4-FGln (^18^F-**1**), a fluorinated analogue of natural L-glutamine, displayed the best uptake properties in tumor cells as compared to the other stereoisomers.

Based on these precedents, we carried out the stereospecific synthesis of 4-FGln (1), to be used both for *in vitro* biological assays and as a reference compound in radio-HPLC analyses.

The synthesis procedure moved upon the previously reported footsteps (14), apart from some optimization variants. Thus, as shown in **Supplemental Figure 1**, starting from commercially available 2*S*-configured protected homoserine **2**, the synthesis path proceeded uneventfully, providing the tosyl product **10** in 29% overall yield. This advanced intermediate was ready for either the subsequent radiofluorination step to [^18^F]4-FGln, or its transformation to “cold” target **1**.

Following the reported procedure for fluorination step of **10** to **11**, we were able to isolate but low amounts of the desired product (32% yield instead of the reported 77%). Thus, we proceeded to slightly modify the procedure by adopting the following conditions: (*i*) TASF (5 equiv) and Et_3_N·(HF)_3_ (3 equiv) till reaching pH 5; (*ii*) solution concentration was a critical parameter and 0.1 M was judged optimal; and (*iii*) dry solvents were necessary to avoid C2 epimerization. Under these conditions, compound **11** was isolated in a very good 86% yield with complete stereochemical integrity.

Finally, global removal of the acid-sensitive protecting groups within **11** was carried out by employing trifluoroacetic acid and dimethyl sulfide, to provide crude fluoroglutamine **1**. The purification procedure of the target compound **1** turned out to be more troublesome than expected. According to the reported procedure, a first column purification step using Dowex 50WX8-200 (H^+^ form) resin followed by recrystallization from EtOH/H_2_O should have provided the final product in a good yield. In our hands, this method did not furnish any precipitate; after resin column, the eluted fraction was purified by reverse phase HPLC (using H_2_O/TFA 0.1% and acetonitrile as eluent mixture), giving an unknown fluorinated product whose mass spectrum (ESI^+^) coincided with that of the target, but the characterization data (^1^H/^13^C/^19^F NMR spectra, HPLC retention time), though similar, did not perfectly matched those reported for the target. Further attempts of purifications of the crude using the same acidic resin and avoiding the HPLC analysis gave, again, unsuccessful results. The ^19^F-NMR analysis performed directly on the fraction eluted from the Dowex, without concentrating it, revealed the presence of several byproducts. Therefore, the purification procedure was modified, and it was found that evaporation of the reaction mixture to eliminate TFA and direct purification *via* reverse-phase HPLC eluting with H_2_O (+0.1% formic acid) and acetonitrile lastly provided the desired fluoroglutamine **1** (41% yield), which was perfectly consistent with the reported data (optical activity, ^1^H/^13^C/^19^F NMR spectra, HPLC retention time, **Figures S2** and **S3**).

It was concluded that the coexistence of many reactive functional groups (amide, carboxylic acid, amine, fluorine) within the small molecule, together with the presence of two stereocenters, renders the purification of this molecule highly challenging, and particular caution to both basic and acidic conditions has to be paid. We may hypothesize that the use of the acidic Dowex resin in the last stage and/or the 5% aqueous ammonia used to elute the product could be responsible for the undesired formation of less polar, cyclic imide product **12** (**Supplemental Figures 1**, **4–5**), possibly deriving from **1** *via* intramolecular dehydration closure. The data collected for the unknown compound are compatible with the structure of putative imide **12**, while its mass spectrum, coinciding with that of target **1**, could be generated by reopening of imide **12** under the mass source conditions.

In conclusion, 4-FGln **1** was obtained in 10% overall yield over six steps from homoserine **2**.

### (2*S*,4*S*)-*tert*-Butyl 2-(*tert*-Butoxycarbonylamino)-4-Hydroxy-5-Oxo-5-(2,4,6-Trimethoxybenzylamino)Pentanoate (9)

The title compound was prepared from compound **8**, thiourea and sodium bicarbonate, according to a reported procedure (*1*). The crude reaction mixture containing a 1:1 mixture of two diastereoisomeric alcohols **9** and **9’** was purified by flash chromatography on silica gel using CH_2_Cl_2_/MeOH as eluent (gradient from 99:1 to 98.5:1.5). Pure product **9** was isolated in a 44% yield as a white solid.

R_f_: 0.5 (EtOAc/hexane 50:50); ${\left[\alpha \right]}_{D}^{25}$ : −29.8 (c = 1.95, MeOH); lit. (*1*) ${\left[\alpha \right]}_{D}^{26}$ : −28.7 (c = 1.06, MeOH).

^1^H-NMR: (400 MHz, CDCl_3_) *δ* 7.27 (bs, *J* = 5.4 Hz, 1H, NH), 6.13 (s, 2H, Ar-H), 5.47 (d, *J* = 7.7 Hz, 1H, NHBoc), 4.8 (bs, s, 1H, OH), 4.55 (dd, *J* = 13.7, 5.8 Hz, 1H, Ar-CH_A_), 4.43 (dd, *J* = 13.7, 5.3 Hz, 1H, Ar-CH_B_), 4.33 (ddd, *J* = 11.6, 7.8, 3.1 Hz, 1H, CH-NHBoc), 4.06 (dd, *J* = 11.7, 2.4 Hz, 1H, CHOH), 3.83 (s, 9H, OCH_3_), 2.14 (ddd, *J* = 14.0, 12.6, 2.6 Hz, 1H, CH_2_), 1.99 (ddd, *J* = 14.0, 11.9, 3.3 Hz, 1H), 1.46 (s, 9H, *t*-Bu), 1.43 (s, 9H, *t*-Bu).

^13^C-NMR: (100 MHz, CDCl_3_) δ 171.8 (C=O), 171.2 (C=O *t*-Bu), 160.9 (Cq), 159.4 (Cq), 157.3 (C=O Boc), 106.5 (Cq), 90.5 (CH), 82.8 (Cq Boc), 80.9 (Cq *t*-Bu), 68.2 (CH), 55.8 (CH3), 55.3 (CH_3_), 50.8 (CH), 39.1 (CH_2_), 31.7 (CH_2_), 28.1 (3C, CH_3_).

### (2*S*,4*R*)-*tert*-Butyl 2-(*Tert*-Butoxycarbonylamino)-4-Fluoro-5-Oxo-5-(2,4,6-Trimethoxybenzylamino)Pentanoate (11)

To a stirred solution of tris(dimethylamino)sulfonium difluorotrimethylsilicate (TASF, 160 mg, 0.582 mmol) in dry DCM/THF (0.7:0.7 ml), under nitrogen atmosphere, Et_3_N·(HF)_3_ (6 μl) was added. After that, tosylate **10** (76 mg, 0.116 mmol) in dry DCM/THF (0.7:0.7 ml) was added to the TASF solution. The reaction mixture was heated to 50°C and kept under inert atmosphere. Some of the solvent was removed by a nitrogen flux in order to have a final volume of 1 ml (final concentration 0.1 M). After 16 h, the oil bath was removed and EtOAc (8 ml) was added. The organic phase was extracted with NaHCO_3_ solution (0.5 M) (1×), water (1×), and brine (1×). The organic phase was dried with MgSO_4_, filtered and evaporated under reduced pressure. The residue was purified by silica gel flash chromatography (EtOAc/Petroleum Ether 20:80 to 30:70) to give product **11** as a white foam (58.3 mg, 86%).

${\left[\alpha \right]}_{D}^{25}$ : +1.7 (*c* = 1.10, MeOH); *lit (1):* ${\left[\alpha \right]}_{D}^{24}$ : +1.7 (*c* = 1.14, MeOH)

^1^H-NMR: (400 MHz, CDCl_3_) *δ* (ppm) 6.70 (bs, 1H, NH), 6.13 (s, 2H, ArH), 5.33 (d, *J* = 7.4 Hz, 1H, NHBoc) 5.01 (ddd, ^2^*J*_HF_ = 49.6 Hz, ^3^*J*_HH_ = 8.9, 3.3 Hz, 1H, CHF), 4.61 (dd, *J* = 13.7, 5.9 Hz, 1H, ArC*H*_A_) 4.40-4.36 (m, 2H, ArCH_B_ + CH_2_), 3.82 (s, 9H, OCH_3_), 2.52 (dddd, ^3^*J*_HF_ = 34.0, *J*_HH_ = 15.0, 5.3, 3.4 Hz, 1H, CH_2A_) 2.22-2.17 (m, 1H, CH_2B_) 1.46 (s, 9H, *t*-Bu) 1.44 (s, 9H, *t-*Bu). ^13^C-NMR: (100 MHz, CDCl_3_) *δ* (ppm) 170.7 (C=O), 168.4 (d, ^2^*J*_CF_ = 19.0 Hz), 161.1 (Cq), 159.3 (Cq), 106.1 (Cq), 90.6 (CH), 90.1 (d, ^1^*J*_CF_ = 186.2 Hz, CF), 55.7 (CH_3_), 55.3 (CH_3_) 35.3 (d, ^2^*J*_CF_ =20.1 Hz, CH_2_), 31.9 (CH_2_), 28.3 (3C, CH_3_), 27.9 (3C, CH_3_). ^19^F-NMR: (376 MHz, CDCl_3_) *δ* (ppm) −189.2 (ddd, *J* = 49.5, 37.0, 19.1 Hz).

### (2*S*,4*R*)-4-Fluoroglutamine (1)

Protected 4-fluoroglutamine **11** (0.104 g, 0.208 mmol) was put in a two-neck flask, kept under N_2_ atmosphere and cooled at 0°C with an ice bath. Dimethylsulfide was added (53 μl, 0.726 mmol), and immediately after TFA was added dropwise (5 ml, 3.53 mmol). The reaction was then removed by the ice bath and kept under stirring for 2.5 h at room temperature. TFA was evaporated, and the crude was washed with DCM (2 × 1 ml) and Et_2_O (2 × 1 ml) removing the solvent with a Pasteur pipette. The crude was purified by a HPLC-RP ([C_18_-10 μm column, 21.2 × 250 mm); solvent A H_2_O (0.1% v/v formic acid); solvent B: ACN; flow rate 8.0 ml/min; detection 210 nm] using the following gradient elution: 0–1 min 0% B, 1–14 min 0–30% B, 14–23 min 30 B, 23–29 min 30–0% B (R_t_ = 11.4 min), obtaining the product 4-F-Gln **1** as a white solid (14 mg, 41%).

${\left[\alpha \right]}_{D}^{25}$ : +45.8 (*c* = 0.15, H_2_O); *lit (1):* ${\left[\alpha \right]}_{D}^{24}$ : +46.2 (*c* = 0.16, H_2_O)

^1^H-NMR: (400 MHz, D_2_O) *δ* (ppm) 5.25 (ddd, ^2^*J*_H-F_ = 48.9 Hz, *J*_H-H_ = 10.2, 2.9 Hz, 1H, CHF), 3.94 (dd, *J* = 6.4, 6.4 Hz, 1H, C*H*NH_2_), 2.56 (dddd, ^3^*J*_H-F_ = 37.6 Hz, ^3^*J*_H-H_ = 15.6, 6.0, 3.0 Hz, 1H, C*H*_A_), 2.33 (dddd, ^3^*J*_H-F_ = 15.8 Hz, *J*_H-H_ = 15.8, 10.3, 7.1 Hz, 1H, C*H*_B_). ^13^C-NMR: (75 MHz, CD_3_OD) *δ* (ppm) 174.8 (d, ^2^*J*_C-F_ = 20.0 Hz), 174.2 (C=O), 90.1 (d, ^1^*J*_C-F_ = 183 Hz, CF), 53.1 (CH), 34.4 (d, ^2^*J*_C-F_ = 20.0 Hz, CH_2_). ^19^F-NMR: (376 MHz, D_2_O) *δ* (ppm) -188.1 (ddd, *J* = 48.9, 37.6, 15.8 Hz). MS (ESI^+^) = 165.06 (M+H)^+^.

### *In Vitro* Characterization of Glutamine and 4-FGln Transport

The human MM cell lines (HMCLs) RPMI8226 and JJN3 were purchased from Leibniz Institute Deutsche Sammlung von Mikroorganismen und Zellkulturen GmbH (Braunschweig, Germany). Cells were seeded in 24-well multidish plates (Falcon, Becton Dickinson Biosciences, Franklin Lakes, NJ, USA) at 750×10^3^ cells/well 48 h before performing [^3^H]-Gln (L-(3,4-^3^H(N))-Gln, PerkinElmer, Groningen, Netherlands), and 4-FGln uptake. Cells were rinsed with Earle’s Balanced Salt Solution (EBSS, NaCl 117 mM, TrisHCl 26 mM; KCl 5.3 mM, CaCl_2_ 1.8 mM, MgSO_4_·7H_2_O 0.81 mM, choline phosphate 0.9 mM, glucose 5.5 mM, supplemented with 0.02% Phenol Red, adjusted at pH 7.4).

For the kinetic analysis of 4-FGln and Gln inhibition on Gln transport, cells were incubated for 30 s at pH 7.4 in EBSS in the presence of [^3^H]-Gln (2 μCi/ml, 0.1 mM) and of increasing concentrations (0.05, 0.25, 1, 5, or 10 mM) of 4-FGln or Gln. At the end of the uptake, cells were rapidly washed with ice-cold urea (300 mM), to block amino acid trans-membrane fluxes, and extracted with absolute ethanol. Extracts were mixed with 200 μl of scintillation fluid and counted with a scintillation spectrometer (Microbeta2, PerkinElmer, Milan, Italy). [^3^H]-Gln influx data, obtained at the different concentrations of the inhibitors, were fit to the equation for competitive inhibition:


$$v=v_{0}-\frac{I_{\max}\cdot [4-\text{FG}\ln]}{{\left[I\right]}_{0.5}+[4-\text{FG}\ln]}$$

For the discrimination of the transporters involved in 4-FGln uptake, cells were incubated for 1 min at pH 7.4 in EBSS in the presence of [^3^H]-Gln (10 μCi/ml, 0.1 mM) or 4-FGln (0.1 mM), and the influx was measured in the absence or in the presence of α-methylaminoisobutyric acid (MeAIB, 5 mM), L-threonine (Thr, 5 mM), or 2-aminobicyclo[2.2.1]heptane-2-carboxylic acid (BCH, 5 mM), as specific or preferential competitive inhibitors of SNAT1/2, ASCT2, or LAT1 transporters, respectively (11). At the end of the uptake, cells were rapidly washed with ice-cold urea (300 mM), and the intracellular amino acid content was extracted with 100 μl of cold absolute ethanol. For cells incubated with radiolabeled Gln, extracts were mixed with 200 μl of scintillation fluid and counted as described above, while the intracellular content of 4-FGln was quantified by high performance liquid chromatography coupled to tandem mass spectrometry (HPLC-MS/MS, see below). For both Gln and 4-FGln, influx data are expressed as pmol/mg prot/min.

### Quantification of 4-FGln Intracellular Content

An Accela HPLC system (Thermo, USA) coupled to a TSQ Quantum Access Max Triple Quadrupole mass spectrometer (Thermo, USA), equipped with a heated electrospray (H-ESI) ion source, was employed for 4-FGln detection. HPLC separation occurred by a linear gradient employing a Phenomenex HILIC Luna column (2.1 × 100 mm; 3 µm particle size; Phenomenex, USA). Eluent A: 90:10 acetonitrile: HCOONH4 1 mM, pH 3.2; eluent B: 50:40:10 acetonitrile: water: HCOONH_4_ 1 mM, pH 3.2). HPLC Gradient was as follows: t=0 min: 90%A; t=0.5 min: 90%A; t=7 min: 50%A; t=8 min: 90%A; with 8 min reconditioning time. Total run time: 16 min. Flow rate: 0.30 ml/min; injection volume: 10 µl. Instrumental parameters were set as follows: ion source voltage: 4,000 V; Capillary temperature: 270°C; sheath gas (N_2_): 35 psi; auxiliary gas (N_2_): 15 psi; collision gas (Ar) pressure: 1.5 mtorr. Mass spectrometer operated in positive ion (ESI+) and in Multiple Reaction Monitoring (MRM) mode. Tube lens voltages (TL) and collision energies (CE) for each parent-product ion transition were optimized by Flow Injection Analysis (FIA). The following transitions were employed: 4-FGln: *m/z* = 165.07 [M+H]^+^→ *m/z* = 148.08, 102.16, 82.20 (TL: 47 V; CE: 5, 16, 22 eV, respectively; Internal Standard: *N*-acetylcysteine *m/z* = 136.04 [M+H]^+^→ *m/z* = 119.07, 90.16, 77.15 (TL: 38 V; CE: 5, 13, 16 eV).

Ethanol cell extracts containing 4-FGln were dried by gentle nitrogen flow and reconstituted in HPLC eluent (90:10 acetonitrile: HCOONH_4_ 1 mM) immediately before HPLC-MS/MS analysis. Calibration curves for 4-FGln were built employing seven calibration standards (CS) in the concentration range 100 nM–10 μM starting from 4-FGln stock solutions in water. Coefficients of correlations (r^2^) were > 0.99 for all curves. The specificity of the method was evaluated by comparison of HPLC-ESI-MS/MS traces of 4-FGln at the Lower Limit of quantification to those of blank cell extracts. Xcalibur software version 2.2 (Thermo, USA) was employed for both data acquisition and processing.

### Radiosynthesis of [^18^F]4-FGln

Radiosynthesis of [^18^F]4-FGln was obtained as described in literature (15), adapting the reported procedure to the in-house modified GE TRACERlab FX Module. [^18^F]Fluoride was produced *via* the ^18^O(p,n)^18^F nuclear reaction by irradiation of [^18^O]H_2_O water in a Niobium target (2 ml) using an 18 MeV cyclotron (18 Twin, IBA RadioPharma Solutions, Louvain-la-Neuve, Belgium). The radionuclide was transferred from target in a water bolus by means of helium flow and trapped in a Chromafix^®^ PS-HCO_3_ cartridge (ABX, Radeberg, Germany) to separate [^18^O]H_2_O. [^18^F]Fluoride was eluted with 1 ml of 18-crown-6 in methanol (8 mg/ml) and 0.18 ml of KHCO_3_ in water (8 mg/ml). Solvents were evaporated by heating to 75°C under reduced pressure. After azeotropic distillation, the precursor *tert*-butyl-(2S,4S)-2-[(tert-butoxycarbonyl)amino]-5-oxo-4-tosyloxy-5-[(2,4,6-trimethoxybenzyl)amino] pentanoate [Tosyl Precursor, compound **10**, **Supplementary Figure 1** (3.5 mg)], dissolved in 0.6 ml of acetonitrile, was added to the dried residue in the reaction vessel. The reaction mixture was heated at 70°C for 15 min without stirring. After quenching by addition of 0.6 ml of water and dilution with 3.5 ml of HPLC solvents (MeOH/H_2_O 75:25+0.1% HCOOH), the reaction mixture was injected in a semipreparative C18 HPLC column (Luna, Phenomenex) for separation of the radiolabeled intermediate (retention time: 18 min) from unreacted [^18^F]Fluoride and other by-products. The intermediate peak was collected in the round bottom flask containing 20 ml of water and loaded on an Oasis HLB cartridge (Waters SPA, Milan, Italy). After washing with water, product was eluted with 1.2 ml of ethanol into the second reaction vessel. The evaporation of the solvent was followed by the addition to the dried residue of 1 ml of trifluoroacetic acid (TFA) containing 20 µl of anisole. Hydrolysis was achieved by heating at 60°C for 5 min, and excess TFA and volatile impurities were removed by evaporation. Radiolabeled product was dissolved in 10 ml of Phosphate Buffered Saline (PBS) and finally recovered in a sterile vial.

### Quality Controls of [^18^F]4-FGln and Stability

Chiral analytical HPLC column Chirex 3126 (Phenomenex, CA, USA) was employed for determination of radiochemical and enantiomeric purity. [^18^F]4-FGln was eluted at 6.5–7.0 min with CuSO_4_ 1 mM at 1 ml/min. pH was measured with indicator strips. The residual anisole in the final solution was quantified by analytical C18 HPLC (Luna, Phenomenex), mobile phase MeOH: H_2_O = 75: 25 + 0.1% HCOOH, flow rate 1 ml/min, λ = 269 nm. The concentration was obtained by fitting to a calibration curve of standard anisole solution. TFA content was obtained by analytical C18 HPLC (Luna, Phenomenex), mobile phase 4.5 mM TBAOH: MeOH = 60: 40 pH 3, flow rate 1 ml/min, λ = 200 nm by fitting to a calibration curve of standard TFA solution. Residual ethanol content was quantified by GC by fitting to a calibration curve of standard ethanol solution.

In-vial stability of [^18^F]4-FGln was evaluated at different time points (1 h, 2 h, 3 h) by chiral HPLC analysis and pH measurements.

### Animal Models and Treatments

Mice were maintained under specific pathogen-free conditions (SPF) in the San Raffaele Scientific Institute facility. Experiments were performed according to national rules and authorized by the Italian Ministry of Health (n. 34/2018-PR).

C57BL/6J mice (6–8 weeks, Charles River Breeding Laboratories, Calco, Italy) were injected intravenously (i.v.). with 0.5 × 10^6^ Vk12598 cells in 200 µl of PBS. Mice were monitored weekly for M-spike and MM progression by retro-orbital bleeding. At weeks 3, 4, and 5, mice underwent PET with [^18^F]FDG and [^18^F]4-FGln on consecutive days.

For xenograft experiments, female NOD.SCID mice (4–6 weeks, Charles River Breeding Laboratories, Calco, Italy) were subcutaneously injected with 5 × 10^6^ JJN3 HMCL in 100 µl of PBS. Tumors were measured twice a week with caliper, and tumor volume was calculated as (a × b^2^)/2; a: long side, b: short side. For evaluation of tracer biodistribution, 6 and 12 days after cell inoculation seven mice underwent PET scans with [^18^F]FDG and [^18^F]4-FGln in consecutive days. An additional group of mice was randomly assigned to two groups: vehicle- (n = 8) and Bortezomib-treated (n = 7). Bortezomib (1 mg/kg) was administered by intravenous injections on d1 and d4. Before (d-1 and d0) and after treatment (d5 and d6), animals underwent PET with [^18^F]FDG and [^18^F]4-FGln and sacrificed on d7. Efficacy was determined according to the adapted RECIST score (16). This index was defined as Tumor Volume Response (TVR) and calculated as the percentage change in median tumor volume measured by caliper at the end of treatment over the median tumor volume before treatment. Treatment response was defined as Partial Response (PR) (TVR > −30%); Stable Disease (SD) (TVR < −30% and < +20%), and Progressive Disease (PD), (TVR > +20%). In this study, PR and SD mice were grouped as responders.

### PET Acquisition

PET studies were performed on a YAP-(S)-PET II (ISE S.r.l., Pisa, Italy) (17). [^18^F]FDG is routinely prepared in our facility for clinical use (European Pharmacopoeia X Edition), whereas [^18^F]4-FGln preparation was described above. [^18^F]FDG (4.32 ± 0.23 MBq) and [^18^F]4-FGln (4.46 ± 0.41 MBq) were injected in a tail vein of fasted mice (>6 h). Dynamic [^18^F]FDG acquisitions started at 60 min post injection and lasted 30 min (six scans of 5 min each) (18, 19). Mice were positioned prone on a “hand-made” mold on the PET bed to maintain the position with the tumor centered in field of view. For [^18^F]4-FGln pilot study, mice (n = 3) underwent a 90-min dynamic acquisition (frames: 4 of 2.5 min, 4 of 5 min, 6 of 10 min each) to determine the optimal imaging time for uptake measures. In the subsequent studies, dynamic PET acquisitions started at 15 min lasting 30 min (six scans of 5 min each). Mice were positioned with the tumor centered in field of view (20) and anesthetized (isoflurane 2%) during acquisition.

### Images Quantification

All images were calibrated with a dedicated phantom, corrected for the radionuclide half-life decay, and then quantified with PMOD 3.2 (Zurich, Switzerland). To set acquisition time and verify reversibility of [^18^F]4-FGln (21), we applied the simplified Logan analyses using muscle as input function (21, 22) to three JJN3 mice. Regions of interest (ROIs) were drawn on tumor and thorax muscle and data expressed as %ID/g to obtain time-activity curves (TACs) of [^18^F]4-FGln. TACs of T/B revealed a time window of 15 to 60 min after injection when the T/B reached stable values. For comparison studies, images were processed as previously described (20), and maximum tumor to muscle ratios (Tmax/M), standardized uptake value (SUV), volume of radioactivity uptake ([^18^F]FDG and [^18^F]4-FGln metabolic tumor volume), total lesion glycolysis (TLG), and total lesion glutaminolysis (TLGln) were calculated. To this aim, PET images were thresholded, as previously validated and described by Krak et al., to create masks for the automatic extraction of the volume of tracer distribution (23). Using Region of Interest (ROI), we first calculated maximum radioligands concentration in tumor (MBq/g) (upper threshold value) and mean radioligands distribution in background (thorax muscle, MBq/g, ROI dimension 0.022 cm^3^) (background value). Then, lower threshold was calculated as the halfway value between upper threshold value and background value. This method allowed to automatically extract the metabolic tumor volume (cm^3^) within of the threshold values and the maximum and mean uptake of the tumor. Thanks to the positioning in the mold, [^18^F]4-FGln images were co-registered with [^18^F]FDG images using PMOD 3.2 and subsequently [^18^F]4-FGln mask of metabolic volume was co-registered to that of [^18^F]FDG and overlapped, so that exclusive radiotracer uptake regions were identified. The profile of backbone visible both with [^18^F]FDG, and [^18^F]4-FGln was used as anatomic landmark and co-registration quality judged and confirmed by an expert in the field (S.V.). For syngeneic model, ROIs were manually drawn on spleen, thorax muscle (0.02 cm^3^), and femur (0.02 cm^3^), and Tmax/M and SUV were calculated.

### Murine Serum Protein Electrophoresis

Blood was collected in Eppendorf by retro-orbital sampling. Semiautomated electrophoresis was performed on the Hydrasys instrument (Sebia, Lissex, France). According to the manufacturer’s instructions, 10 μl of undiluted serum were manually applied to the Hydragel agarose gels (Sebia). The subsequent steps, electrophoresis (pH 9.2, 20 W constant current at 20°C), drying, amidoblack staining, de-staining, and final drying, were carried out automatically. The use of Hydrasys densitometer and Phoresis software (Sebia) for scanning resulting profiles provided accurate relative concentrations (percentage) of individual protein zones. M-spike levels were calculated as total gamma globulins/albumin ratio (G/A).

### Western Blot of ASCT2 in Murine MM

BM samples of either Vk12598-injected or non-injected C57BL/6J mice were lysed in RIPA buffer (Cell Signaling Technology). Immunoblotting was performed as previously described (11). Anti-ASCT2 (rabbit, polyclonal, 1:1,000, Cell Signaling Technology) and anti-β-tubulin (mouse, polyclonal, 1:1,000, Sigma) were used as primary antibodies.

### Statistics

*In vivo* data are presented as means ± standard deviation (SD). All statistical analyses were performed using Prism 8 (GraphPad Software, Inc., USA). Statistics are detailed in figure legends. Differences were considered significant when p < 0.05.

## Results

### Chemical and Radiochemical Syntheses

Based on previously reported procedures (14), we carried out the stereospecific synthesis of 4-FGln (**Supplementary Figures 1–5**) to be used both for *in vitro* biological assays and as a reference compound in radio-HPLC analyses. This section as well as radiolabeling have been extensively described in *Methods*. For radiochemical synthesis, 2.5 ± 1.4 GBq of [^18^F]4-FGln were obtained in about 100 min by automated synthesis [see above, *(2S,4R)-4-Floroglutamine (1)*]. Radiochemical yield was 12.3 ± 5.4% n.d.c (n=16). Radiochemical purity was 98.9 ± 0.9%, and stereochemical purity was 95 ± 3.5%. The pH of the solution was 6.9–7. Residual solvents are below the admitted limit (Anisole 0.3 ± 0.1 μg/ml, TFA 78.6 ± 60 μg/ml, Ethanol 2.5 ± 0.4 mg/ml).

### Characterization of 4-FGln Uptake

**Figure 1:**
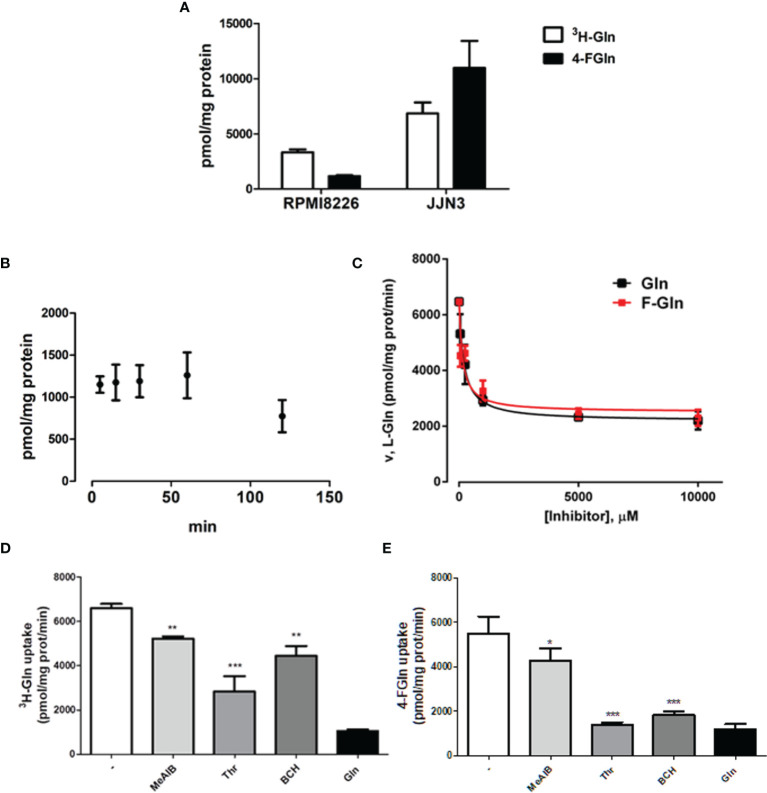
Gln and F-Gln accumulation and uptake in MM cells. **(A)** RPMI8226 and JJN3 cells were incubated in EBSS containing L-[3,4-^3^H(N)]-Gln (0.1 mM, 2 µCi/ml, ^3^H-Gln) or 4-FGln (0.1 mM) for 5 min. Means ± SD of two experiments with five independent determinations each are shown. **(B)** RPMI8226 cells were incubated in EBSS containing 4-FGln (0.1 mM) for 5, 15, 30, 60, and 120 min. Means ± SD of two experiments with five independent determinations each are shown. **(C)** RPMI8226 cells were incubated for 30 s in EBSS containing L-[3,4-^3^H(N)]-Gln (0.1 mM, 2 µCi/ml) in the presence of increasing doses (0.050, 0.25, 1, 5, or 10 mM) of cold 4-FGln or Gln. Means ± SD of two experiments with five independent determinations each are shown. **(D–E)** 1-min 4-FGln **(D)** or Gln **(E)** uptake in RPMI8226 cells in the presence of the indicated inhibitors, used at 5 mM. Means ± SD of two experiments with five independent determinations each are shown. *p < 0.05, **p < 0.01, ***p < 0.001. as assessed with a two-tailed t test.

Gln and 4-FGln were rapidly accumulated in either RPMI8226 or JJN3 HMCLs, reaching a maximum at 5 min of incubation (**Figure 1A**). 4-FGln accumulation remained fairly stable up to 60 min, while showed a partial decrease at 120 min (**Figure 1B**). To verify if 4-FGln enters MM cells through the same transporters used by Gln, radiolabeled Gln uptake was performed in the presence of increasing doses of either 4-FGln or Gln in RPMI8226. As shown in **Figure 1C**, 4-FGln inhibited [^3^H]-Gln with a potency comparable to that of the natural amino acid (4-FGln [I]0.5 = 143 ± 35 µM; Gln [I]0.5 = 193 ± 33 µM). The maximal inhibition was around 70% of the total control uptake for both Gln and 4-FGln. Then, the 1-min influx of 4-FGln (0.1 mM) and [^3^H]-Gln (0.1 mM, 2 µCi/mL) was determined in the presence of MeAIB, a specific substrate of System A carriers, Thr, a preferential substrate of the ASCT2 transporter, or BCH, a substrate of System L transporters. Although determined with different analytical techniques, the uninhibited uptakes of Gln and 4-FGln were fairly comparable (**Figures 1D–E**). Moreover, all the inhibitors tested were able to hinder both 4-FGln and Gln uptake, with Thr showing the highest and MeAIB the lowest inhibitory activity (**Figures 1D–E**). BCH inhibited more 4-FGln than Gln uptake, suggesting that 4-FGln exploits sodium-independent system L transporters more than the natural amino acid.

### Tumor and Muscle Time Activity Curves of [^18^F]4-FGln

**Figure 2:**
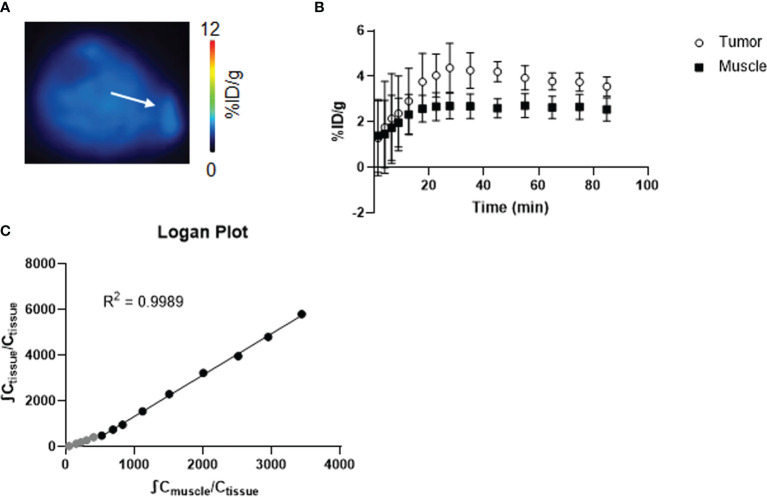
Kinetic study of [^18^F]4-FGln. **(A)** Coronal PET image summed from 15 to 50 min of dynamic scan of a representative JJN3 tumor-bearing mouse expressed as %ID/g. The white arrow indicates tumor. **(B)** Time-activity curves of the muscle and tumor of JJN3 tumor-bearing mice (n = 3). **(C)** Logan graphical analysis of TACs of JJN3 tumor-bearing mice. Data points used for the graphical fits are denoted in black, and unused data points are gray.

Firstly, we characterized [^18^F]4-FGln kinetics *in vivo* using the NOD.SCID JJN3 xenograft model (**Figure 2**). **Figure 2A** shows JJN3 tumor and muscle TACs. In tumor, [^18^F]4-FGln uptake peaked at 25 min slowly declining thereafter (**Figure 2B**). The Logan plot (**Figure 2C**) confirmed linearity starting at 15 min from injection, consistent with largely reversible tracer exchange (21). The R^2^ value for the fits of data from JJN3-grafted mice was 0.9989. For this reason, acquisition time was set between 15 and 45 min p.i. for subsequent studies.

### *In Vivo* [^18^F]4-FGln and [^18^F]FDG Distribution in MM Models

[^18^F]4-FGln and [^18^F]FDG were compared in two MM models: a syngeneic model (murine Vk12598 cells) and a xenograft model (JJN3 cells).

**Figure 3:**
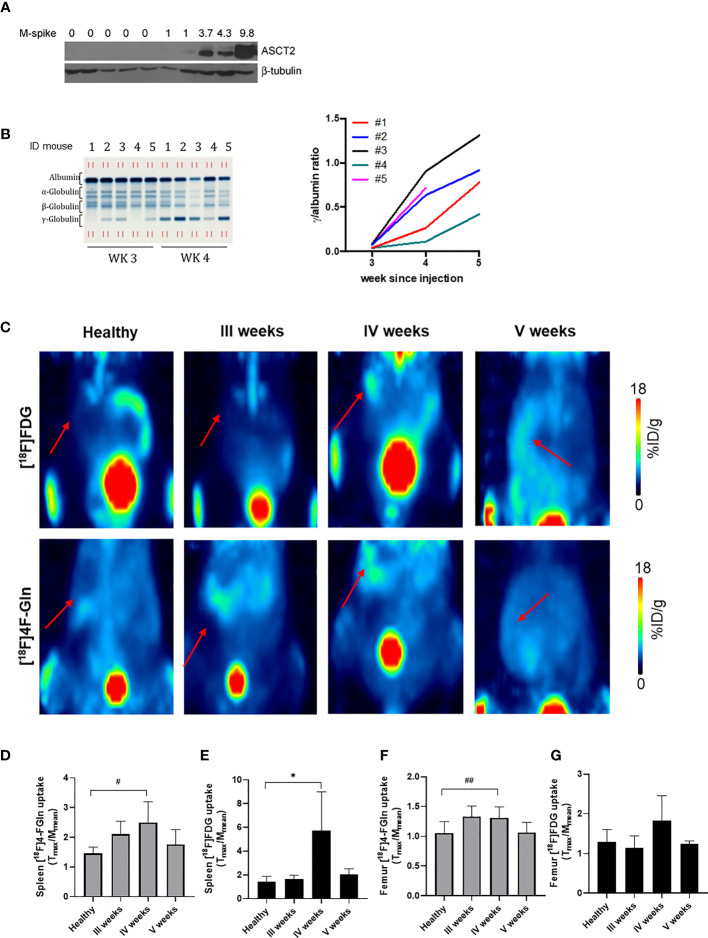
Radiotracers distribution in a syngeneic MM model. **(A)** ASCT2 expression in the bone marrow (femurs) of control (lanes 1–5) and Vk12598 injected mice 5 weeks after injection (lanes 5–10). Numbers indicate the M-spike level at the time of the analysis. **(B)** Representative serum protein electrophoresis gel (left) and tumor growth curves represented as total gamma/albumin ratio (right) of mice from 1 to 5 measured at 3, 4, and 5 weeks after Vk12598 injection. **(C)** Representative maximum intensity projection (MIP) images of from [^18^F]FDG and [^18^F]4-FGln of a healthy mouse and at III, IV, and V weeks from the injection of Vk12598 cells. The red arrows indicate the spleen. **(D, E)** Quantitative analysis of radiotracers uptake expressed as T/M in the spleen. Differences intragroups were tested for significance using paired t test. *p = 0.03; ^#^p = 0.04. **(F, G)** Quantitative analysis of radiotracers uptake expressed as T/M in the femur. Differences intragroups were tested for significance using paired t test. ^##^p = 0.002.

Upon injection into C57BL/6 mice, Vk12598 cells colonize the BM, without lytic lesions, and the spleen, generating an aggressive MM (24). The expression of the glutamine transporter ASCT2 was checked in the BM (femur) of Vk12598 MM bearing mice. As shown in **Figure 3A**, the expression of the transporter increased along with MM progression, monitored with M-spike in blood samples collected at weeks 3, 4, and 5 after cell injection (**Figure 3B**) (25). Uptake of both tracers was measurable in the spleen of MM mice by week 3, reached a peak at week 4 [[^18^F]4-FGln (T/M: 2.51 ± 0.69); [^18^F]FDG (T/M: 5.73 ± 3.26)] and declined thereafter (**Figures 3C–E**). At the 4th week, T/M values of [^18^F]FDG were definitely higher than that of [^18^F]4-FGln. We performed also a quantification of the femur uptake. Healthy mice displayed a similar uptake of both radiotracers, whereas at the 4th week we observed a significant increase of [^18^F]4-FGln uptake. The uptake of [^18^F]FDG was more heterogeneous (**Figures 3F, G**).

**Figure 4:**
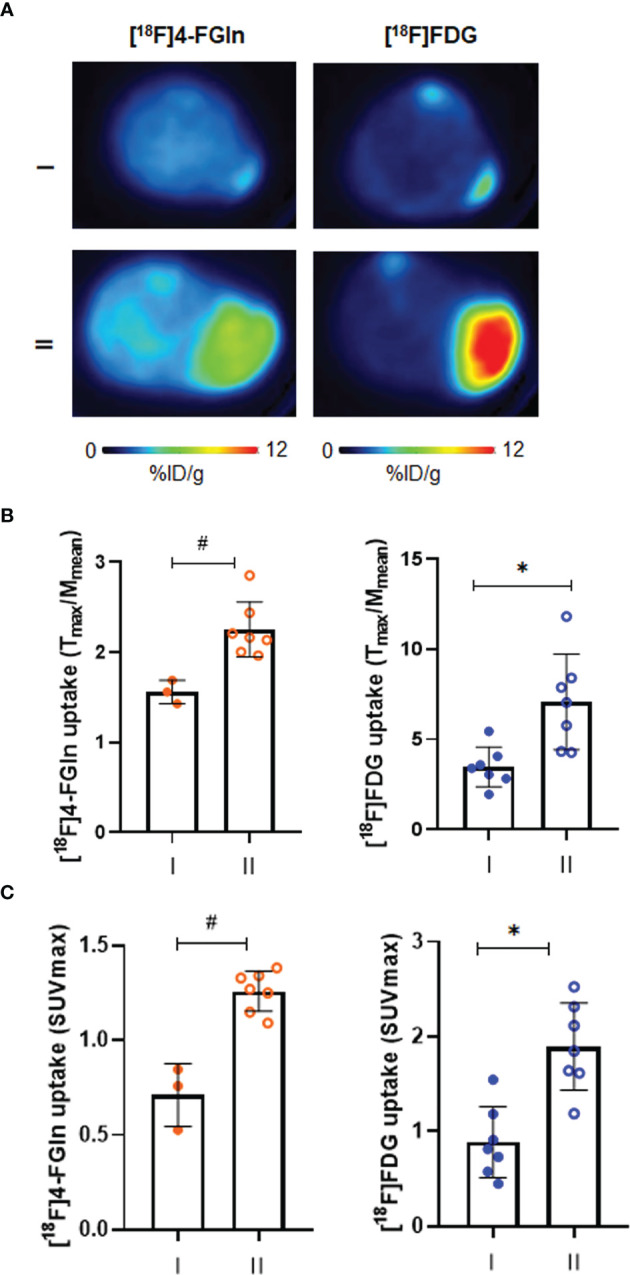
Characterization of [^18^F]FDG and [^18^F]4-FGln uptake in JJN3 tumor model. **(A)** Transaxial [^18^F]FDG and [^18^F]4-FGln PET images of a representative JJN3 tumor-bearing mouse acquired on days 5–6 (I) and 12–13 (II) expressed as %ID/g. **(B)** Quantitative analysis of radiotracers uptake expressed as T/M. Differences intra- and intergroups were tested for significance using Wilcoxon matched-pairs signed rank-test and Unpaired Mann-Whitney test. *p = 0.0156; ^#^p = 0.0167. **(C)** Quantitative analysis of radiotracers uptake expressed as SUVmax. Differences intra- and intergroups were tested for significance using Wilcoxon matched-pairs signed rank-test and Unpaired Mann-Whitney test. *p = 0.0156; ^#^p = 0.0167.

In the JJN3 cells model, mice underwent a first scan at the tumor size of 72.8 ± 35.95 mm^3^, displaying both [^18^F]4-FGln (T/M: 1.6 ± 0.1) and [^18^F]FDG (T/M: 3.5 ± 1.1) uptake. Tumor volume (896.8 ± 349.00 mm^3^), [^18^F]4-FGln uptake, and [^18^F]FDG uptake (T/M: 2.3 ± 0.3 and 7.1 ± 2.6, respectively) increased after 1 week (**Figures 4A, B**). In any case, [^18^F]FDG displayed a higher uptake than [^18^F]4-FGln. Similar results were obtained considering SUVmax (**Figure 4C**).

### *In Vivo* [^18^F]4-FGln and [^18^F]FDG Displayed Different Response After Bortezomib Administration in Mice

**Figure 5:**
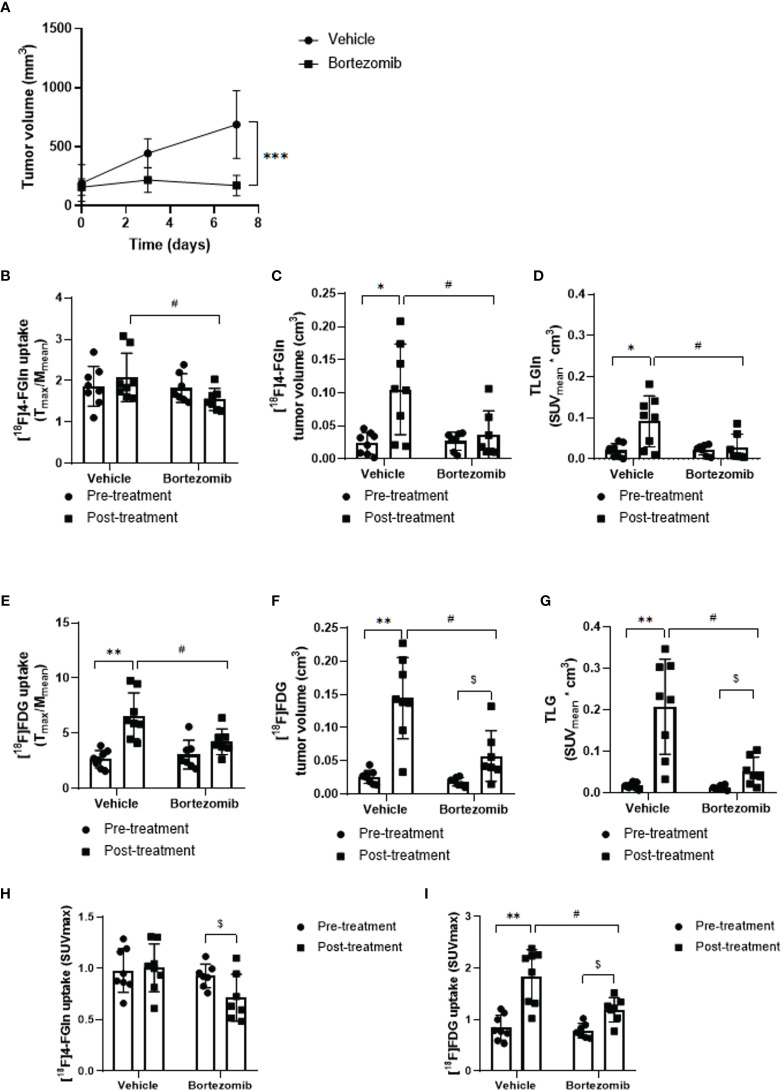
Effect of Bortezomib treatment on [^18^F]FDG and [^18^F]4-FGln uptake measured by PET. **(A)** Tumor volume measured by caliper in vehicle and Bortezomib-treated mice. Data are expressed as mean ± SD and were analyzed by Unpaired Mann-Whitney test. ***p = 0.0006. **(B, E)** Quantitative analysis of [^18^F]FDG and [^18^F]4-FGln T/M uptake ratio before and after treatment with vehicle and Bortezomib. **(C, F)** Volumetric analysis of [^18^F]FDG and [^18^F]4-FGln uptake within the tumor region, pre- and post-therapy. **(D, G)** Quantification of total lesion glycolysis (TLG) and total lesion glutamine (TLGln) within the tumor region, pre- and post-therapy. Differences intra- and intergroups were tested for significance using Wilcoxon matched-pairs signed rank-test and Unpaired Mann-Whitney test. *, ^#, $,^ p < 0.05; **p < 0.01. **(H)** Quantitative analysis of [^18^F]4-FGln and **(I)** [^18^F]FDG SUVmax uptake before and after treatment with vehicle and Bortezomib. Differences intra- and intergroups were tested for significance using Wilcoxon matched-pairs signed rank-test and Unpaired Mann-Whitney test. ^#, $,^ p < 0.05; **p < 0.01.

Administration of Bortezomib to JJN3-challenged mice caused a dramatic reduction in tumor growth when compared to vehicle-treated MM mice (172.0 ± 86.6 *vs* 687.9 ± 286.5 mm^3^; p = 0.0006) (**Figure 5A**). In vehicle-treated mice at day 6, PET demonstrated only a positive, not significant trend of [^18^F]4-FGln uptake (T/M pre: 1.86 ± 0.48, post: 2.08 ± 0.59; p > 0.05), but a significant increase of [^18^F]4-FGln-derived tumor volume (pre: 0.024 ± 0.016 cm^3^, post: 0.105 ± 0.069 cm^3^; p = 0.016) and total lesion glutamine (pre: 0.021 ± 0.016, post: 0.092 ± 0.061; p = 0.016) (**Figures 5B–D**). In parallel, we observed a significant increase of [^18^F]FDG uptake (pre: 2.66 ± 0.78, post: 6.58 ± 2.08; p = 0.0078), [^18^F]FDG-derived tumor volume (pre: 0.026 ± 0.010 cm^3^, post: 0.144 ± 0.061 cm^3^; p = 0.008) and TLG (pre: 0.017 ± 0.007, post: 0.208 ± 0.114; p = 0.008) (**Figures 5E–G**). In Bortezomib-treated animals, we did not observe any increase of [^18^F]4-FGln uptake (T/M), [^18^F]4-FGln metabolic tumor volume, and TLGln after treatment (**Figures 5B–D**). In addition, SUVmax values highlighted a significant reduction of [^18^F]4-FGln (**Figure 5H**). On the contrary, using [^18^F]FDG we observed a significant increase of the radiotracer tumor volume (pre: 0.02 ± 0.006 cm^3^, post: 0.06 ± 0.04 cm^3^; p = 0.016), TLG (pre: 0.01 ± 0.005, post: 0.05 ± 0.03; p = 0.016) (**Figures 5F, G**), and also SUVmax (pre: 0.79 ± 0.14, post: 1.19 ± 0.24; p = 0.016) (**Figure 5I**). For both radiotracers and for all the considered parameters, Bortezomib-treated mice displayed significant lower values than vehicle-treated mice (**Figures 5B–G**).

**Figure 6:**
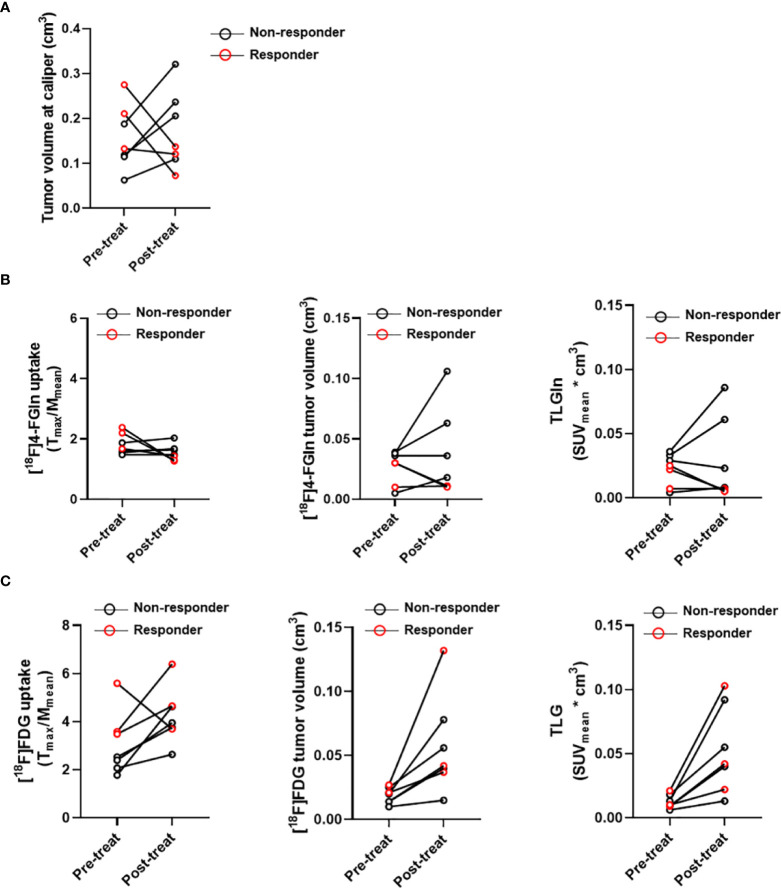
Case by case analysis of “responders” and “non-responders.” **(A)** Tumor volume measured at caliper, **(B)** T/M ratios and tumor volume relative to [^18^F]4-FGln uptake and TLGln, and **(C)** T/M ratios and tumor volume relative to [^18^F]FDG uptake and TLG pre- and post-treatment with Bortezomib in responder (red circles) and non-responder cases (black circles).

To verify if metabolic modifications were related with tumor response, mice in the Bortezomib-treated group were classified as responders and non-responders based upon the adapted RECIST score (**Figure 6A**). Three Bortezomib-treated mice were classified as responders (#7G, #10G, #5G). These mice displayed a reduction of [^18^F]4-FGln T/M ratio, [^18^F]4-FGln-related tumor volume, and TLGln (**Figure 6B**). On the contrary, independently from the response, all mice displayed increased [^18^F]FDG parameters (**Figure 6C**). These results indicate that [^18^F]4-FGln better highlights MM response to Bortezomib than [^18^F]FDG.

**Figure 7:**
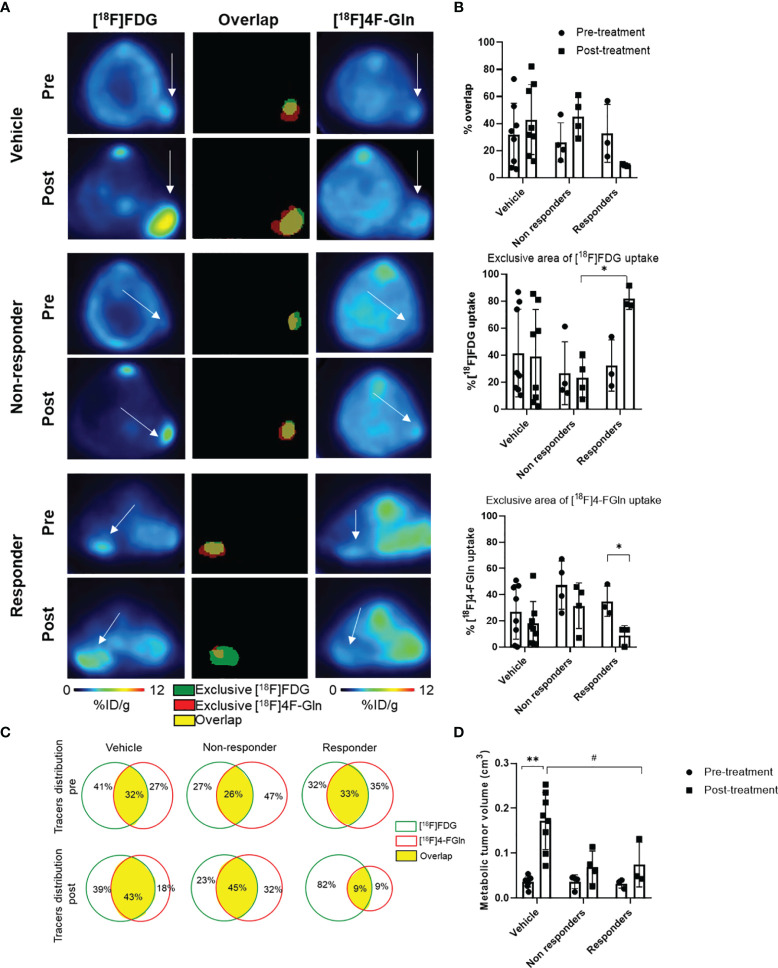
Volumetric analysis defines the distribution of tracers in the tumor and highlights specific therapy-induced alterations in the uptake of individual tracers. **(A)** representative transaxial PET images from [^18^F]FDG and [^18^F]4-FGln and the overlay image of [^18^F]FDG and [^18^F]4-FGln masks in the same mice (vehicle, Bortezomib-non-responder and responder) pre- and post-treatment. White arrows indicate tumor. In overlay images, green color represents exclusive [^18^F]FDG uptake, red color represents exclusive [^18^F]4-FGln uptake, and yellow color represents co-distribution of both radiopharmaceuticals. **(B)** % of overlap, exclusive [^18^F]FDG and [^18^F]4-FGln tracer uptake volumes in vehicle- and Bortezomib-treated responder and non-responder groups. Differences intra- and intergroups were tested for significance using one-way ANOVA with multiple comparisons corrected with Tukey’s test and Wilcoxon matched pairs signed rank test. *p < 0.05. **(C)** Quantitative analysis of single tracers uptake and total distribution pre-and post-treatment in vehicle- and Bortezomib-treated non-responder and responder groups; the green lines represent [^18^F]FDG unique area, red lines represent [^18^F]4-FGln unique area, and the yellow filled space is the overlap of both tracers. **(D)** Metabolic tumor volume (sum of [^18^F]FDG and [^18^F]4-FGln volume of biodistribution) pre- and post-treatment with vehicle and Bortezomib in responders and non-responders. Differences intra- and intergroups were tested for significance using one-way ANOVA with multiple comparisons corrected with Tukey’s test and Wilcoxon matched pairs signed rank test. ^#^p = 0.027; **p = 0.008.

The quantitative volumetric analysis of exclusive areas of [^18^F]FDG and [^18^F]4-FGln uptake, as well as of regions of tracer overlap, was performed (**Figures 7A, B**). This strategy was applied to analyze *in vivo*, in the same animals, the modification in the volume of distribution of the two radiotracers and the presence of regional treatment-related differences in metabolism. Overlapping regions showed the part of the tumor with cells that are both glycolytic and glutaminolytic, while in the other regions one of the two types of metabolism is prevalent. The total metabolic tumor volume (the sum of [^18^F]FDG and [^18^F]4-FGln) increased in all groups (**Figure 7D**). The tracer overlap increased from 32 to 43% after 6 days in control and from 26 to 45% in non-responder mice. On the contrary, the area of tracer overlap decreased at about 9% in responder mice. After 6 days, the area of exclusive [^18^F]4-FGln and [^18^F]FDG uptake of vehicle-treated and non-responder mice remained fairly stable. Conversely, the area of relative exclusive uptake of [^18^F]4-FGln significantly decreased at 9% (p = 0.04) in responders compared to 32% in non-responders, while that of [^18^F]FDG uptake significantly increased at 82% (p = 0.04) compared to 23% in non-responders (**Figures 7B, C**).

## Discussion

Currently, [^18^F]FDG is the standard tracer for the PET/CT scan in MM patients, and the role of this imaging is well established for checking skeletal involvement and extramedullary disease. Recently, the prognostic role of [^18^F]FDG has been further highlighted (2, 26, 27). However, [^18^F]FDG in CT/PET scans may have some limitations due to the presence of MM patients negative but with the presence of the disease. Moreover, [^18^F]FDG carries its own limitations as a radiopharmaceutical, including a rather poor sensitivity for the detection of diffuse bone marrow infiltration, a relatively low specificity, and the lack of widely applied, established criteria for image interpretation (4, 5). Overall, this evidence have led to the development of alternative PET tracers, such as choline and methionine (6, 28), with promising results regarding MM detection. Other tracers are under evaluation (29).

Growing evidence suggests the possible use of [^18^F]4-FGln as a new tracer in cancer patients taking advantage by the Gln-dependent metabolic profile of several tumors. Some preclinical and clinical studies have been published on *in vivo* testing of [^18^F]4-FGln (8, 9, 21, 30–32), supporting its possible use as imaging biomarker. Moreover, [^18^F]4-FGln could be used to study the metabolic profile and the Gln addiction of MM cells in order to design a metabolic-based therapeutic approach.

Recently, we have demonstrated that MM cells are strictly Gln addicted and lack of a sizable expression of the enzyme Glutamine Synthetase (11). Therefore, MM cells only rely on extracellular Gln. Consequently, MM cells are endowed with fast Gln uptake due to high expression of at least three different types of Gln transporters and, in particular, of the sodium-dependent carrier ASCT2, which is overexpressed in several types of Gln-dependent cancers (33). Moreover, we have also recently shown that the massive Gln uptake by MM cells decreases Gln concentration in the tumor microenvironment, contributing to the bone remodeling process induced by MM cells (34). Overall, this evidence provides a rational basis to exploit Gln metabolism in the design of a PET tracer for MM diagnosis and patient stratification.

Here, the possible role of [^18^F]4-FGln as radiotracer for imaging in MM has been evaluated either *in vitro* or *in vivo*. First, we assessed 4-FGln transport *in vitro*, showing that 4-FGln is a reliable Gln transport analogue in human MM cell lines, with a potency as an inhibitor of Gln even larger than the natural amino acid. More importantly, most of the uptake of 4-FGln by MM cells occurs through the ASCT2 transporter, the same carrier exploited preferentially by Gln.

Applicability of [^18^F]4-FGln to *in vivo* studies was confirmed in two models: the well-established syngeneic model, based on the inoculation of murine MM cells Vk12598 and extensively used to provide the rationale for novel therapeutic intervention (24, 25, 35), and a xenograft model based on human JJN3 cells. Our data suggest that in Vk12598 model both [^18^F]4-FGln and [^18^F]FDG can be used to follow disease progression, as indicated by M-spike measurement, in sites of extramedullary disease, despite higher uptake of [^18^F]FDG in extramedullary lesion in comparison with [^18^F]4-FGln, as shown by ROIs analysis. MM can develop disease also in bone marrow, but one possible limit of [^18^F]4-FGln for the detection of medullary disease is the high uptake found in the bone after 60 min from injection (36). In our study, with the signal acquired between 15 and 45 min, bone signal was not evident. To better understand this issue, we analyzed femur uptake in the syngeneic model. We performed a quantification of uptake in femur of the syngeneic mice where bone signal could be increased also by the presence of myeloma. We did not observe any significant difference between the two radiotracers when myeloma was not present (healthy condition). However, at the 4th week we observed a significant increase of [^18^F]4-FGln whereas the uptake of [^18^F]FDG was more heterogeneous. Unfortunately, due to the low sensitivity of the PET system used for the acquisition with our PET system, it is not possible to distinguish the uptake between bone and bone marrow. Further analyses are needed to address this issue.

Also the xenograft model demonstrated the capacity of [^18^F]4-FGln and [^18^F]FDG to be taken up by subcutaneous plasmocytomas. Interestingly, in this model, we observed minimal radiotracer trapping and largely reversible tracer exchange despite the high glutaminase (GLS) activity of the JJN3 line (11) that should lead to F-Gln hydrolysis. On the other hand, [^18^F]4-FGln uptake could reflect glutamine pool rather than GLS activity (8). Indeed, [^18^F]4-FGln tumor kinetics reflects Logan plot confirming the presence of a reversible tracer exchange also in this MM model. Although uptake of [^18^F]4-FGln was lower than [^18^F]FDG uptake also in JJN3 tumors, as observed in the Vk12598 model, variability of [^18^F]4-FGln was remarkably smaller.

To better define the role of [^18^F]4-FGln as radiopharmaceutical, we evaluated the effect of Bortezomib. Our most intriguing finding was that [^18^F]4-FGln and [^18^F]FDG differently perceived the metabolic changes imposed to the tumor by Bortezomib. Indeed, independently from response, Bortezomib increased [^18^F]FDG uptake and volume of distribution, whereas [^18^F]4-FGln parameters were more closely associated with the response, suggesting that [^18^F]4-FGln based PET scan reliably delineates tumor sensitivity to the drug. In line with our observations, it has been reported that [^18^F]FDG uptake may persist in MM patients who achieved complete response to Bortezomib (37). The drug has been reported to stimulate aerobic glycolysis, thus possibly indicating that changes in tumor volume are masked by increased glycolytic flux (38–40). Overall, we showed a marked effect of Bortezomib on tumor cell metabolic phenotype, as indicated by the reduction of volume of exclusive [^18^F]4-FGln uptake in responder mice.

Previous studies suggested that [^18^F]4-FGln labels the intracellular Gln pool and, indirectly, is a glutaminolytic marker (8). This was recently supported by a kinetic analysis applied on murine breast cancer models (21) and confirmed here by the demonstration that [^18^F]4-FGln uptake in JJN3 tumors is described by a one-compartment, reversible tissue model with no trapping. Thus, [^18^F]4-FGln proves a dynamic tool for research and potential clinical use in MM. Moreover, Bortezomib caused a significant reduction of volume of exclusive [^18^F]4-FGln uptake in responder mice, indicating a drug effect on tumor metabolic phenotype. These results suggest that glutaminolysis impairment represents a major factor in Bortezomib efficacy. This confirms the metabolic heterogeneity of MM and suggests that modification in glutaminolysis represents a major event in Bortezomib efficacy. For the reasons above, our results suggest that [^18^F]4-FGln may be a promising radiopharmaceutical for PET molecular imaging of the outcome of metabolically based target therapy acting on glutaminolysis.

Metabolic changes parallel MM progression (41). For instance, glutamine-dependent anaplerosis of the TCA cycle increases from MGUS to myeloma (12). Moreover, a sizable degree of metabolic heterogeneity may be present in the same MM (42), suggesting that subpopulations of MM cells may respond in a different way to therapeutic treatments. For these reasons, the combined use of distinct metabolically related probes, such as [^18^F]4-FGln and [^18^F]FDG, may yield clinically important information.

In conclusion, our data indicate that [^18^F]4-FGln may be a new tracer to detect MM cells in preclinical *in vivo* models. [^18^F]4-FGln might help to explore the potential use of PET to better define the metabolic phenotype of the tumor and the modifications induced by therapy, particularly as a potential marker of treatment response to proteasome inhibitors. Moreover the *in vivo* study of the metabolic profile of myeloma cells by [^18^F]4-FGln could be useful to design future metabolic-based therapeutic approach and for the clinical management of MM patients.

## Data Availability Statement

The original contributions presented in the study are included in the article/**Supplementary Material**. Further inquiries can be directed to the corresponding authors.

## Ethics Statement

The animal study was reviewed and approved by Italian Ministry of Health (n. 34/2018-PR).

## Author Contributions

All authors contributed to the study conception and design. Material preparation, data collection, and analysis were performed by SV, DT, MC, AS, AC, AB, GT, MG, FV, FZ, MB, RM, OB, and NG. The first draft of the manuscript was written by NG and DT, and all authors commented on previous versions of the manuscript. All authors read and approved the final manuscript.

## Funding

The research leading to these results has received funding from: Associazione Italiana per la Ricerca sul Cancro (AIRC) under IG IG2017 ID. 20299 project and International Myeloma Society (IMS) under “Paula and Rodeger Riney Foundation Translational Research Grant” (PI NG) and AIRC under IG 2018, ID. 210808 project (PI BM). The Italian Ministry for Education and Research (MIUR) is gratefully acknowledged for yearly FOE funding to the Euro-BioImaging Multi-Modal Molecular Imaging Italian Node (MMMI). Translational Research Program Grant ID 6618-21 (PI BM) from the Leukemia & Lymphoma Society (L&LS).

## Conflict of Interest

NG received research funding and honoraria from Bristol Mayers Squibb, Celgene, Millenium Pharmaceutical, GSK, Takeda, and Janssen Pharmaceutical.

The remaining authors declare that the research was conducted in the absence of any commercial or financial relationships that could be construed as a potential conflict of interest.

## Publisher’s Note

All claims expressed in this article are solely those of the authors and do not necessarily represent those of their affiliated organizations, or those of the publisher, the editors and the reviewers. Any product that may be evaluated in this article, or claim that may be made by its manufacturer, is not guaranteed or endorsed by the publisher.
