# Supplementary material for: [18F](2S,4R)-4-Fluoroglutamine as a New Positron Emission Tomography Tracer in Myeloma
Source: Front Oncol. 2021 Oct 12;11:760732. doi: 10.3389/fonc.2021.760732 (PMC8546185; doi:10.3389/fonc.2021.760732)

**Title: [^18^F](2*S*,4*R*)-4-Fluoroglutamine as a new Positron Emission Tomography tracer in Myeloma**

Supplementary Material

# Chemistry. General Experimental Notes

Unless otherwise noted, all reactions were performed in oven-dried glassware under an atmosphere of nitrogen or argon. Air-sensitive reagents and solutions were transferred via syringe or cannula and were introduced to the apparatus through rubber septa.

The solvents were according to standard procedures (dichloromethane (ACS grade), was distilled on CaH_2_, THF on Na and benzophenone). Solvents for flash chromatography and filtration including hexane, cyclohexane, ethyl acetate, petroleum ether, DMF, acetone, anhydrous ethanol, methanol and 2-propanol were ACS or HPLC grade and used as received.

Analytical thin layer chromatography (TLC) was performed on silica gel 60 F254 pre-coated plates with visualization under short-wavelength UV light (254 nm) and by dipping the plates with either molybdate reagent (aqueous H_2_SO_4_ solution of ceric sulphate/ammonium molybdate) or ethanol solution of ninhydrin (0.2%), followed by heating. Flash column chromatography was performed using 40-63 μm silica gel using the indicated solvent mixtures. HPLC purifications were performed on a Prostar 210 apparatus equipped with C_18_-10 μm column (Discovery BIO Wide Pore 10 x 250 mm or 21.2 x 250 mm). Optical rotation data [α]_D_ were obtained on a digital polarimeter at ambient temperature using a 100 mm cell with 1 mL capacity and are given in units of 10^-1^ deg·cm^2^·g^-1^. NMR spectra were recorded on Bruker AV400 spectrometer. Chemical shifts (δ) are reported in parts per million (ppm) and referenced to the signal of the lock solvent. Multiplicities are indicated as s (singlet), d (doublet), t (triplet), q (quartet), dd (double doublet), td (triple of doublets), m (multiplet), and b (broad). Coupling constants, *J*, are reported in Hertz. ESI-mass spectra were recorded on API 150EX apparatus and are reported in the form of (*m/z*).

**Supplementary figures and legends**

**Supplementary Figure 1: Synthesis path to advanced tosyl intermediate 10 and (2*S*,4*R*)-4-fluoroglutamine (4-FGln, 1)**. Abbreviations: Boc= tert-butoxycarbonyl; t-Bu= tert-butyl; DMP= Dess-Martin periodinane; Tmob= 2,4,6-trimethoxybenzyl; TASF= tris(dimethylamino)sulfonium difluorotrimethylsilicate; Ts= p-toluensulfonyl.

**Supplementary Figure 2:** ^1^H NMR spectrum (400 MHz, D_2_O) of compound 4-FGln **1**.


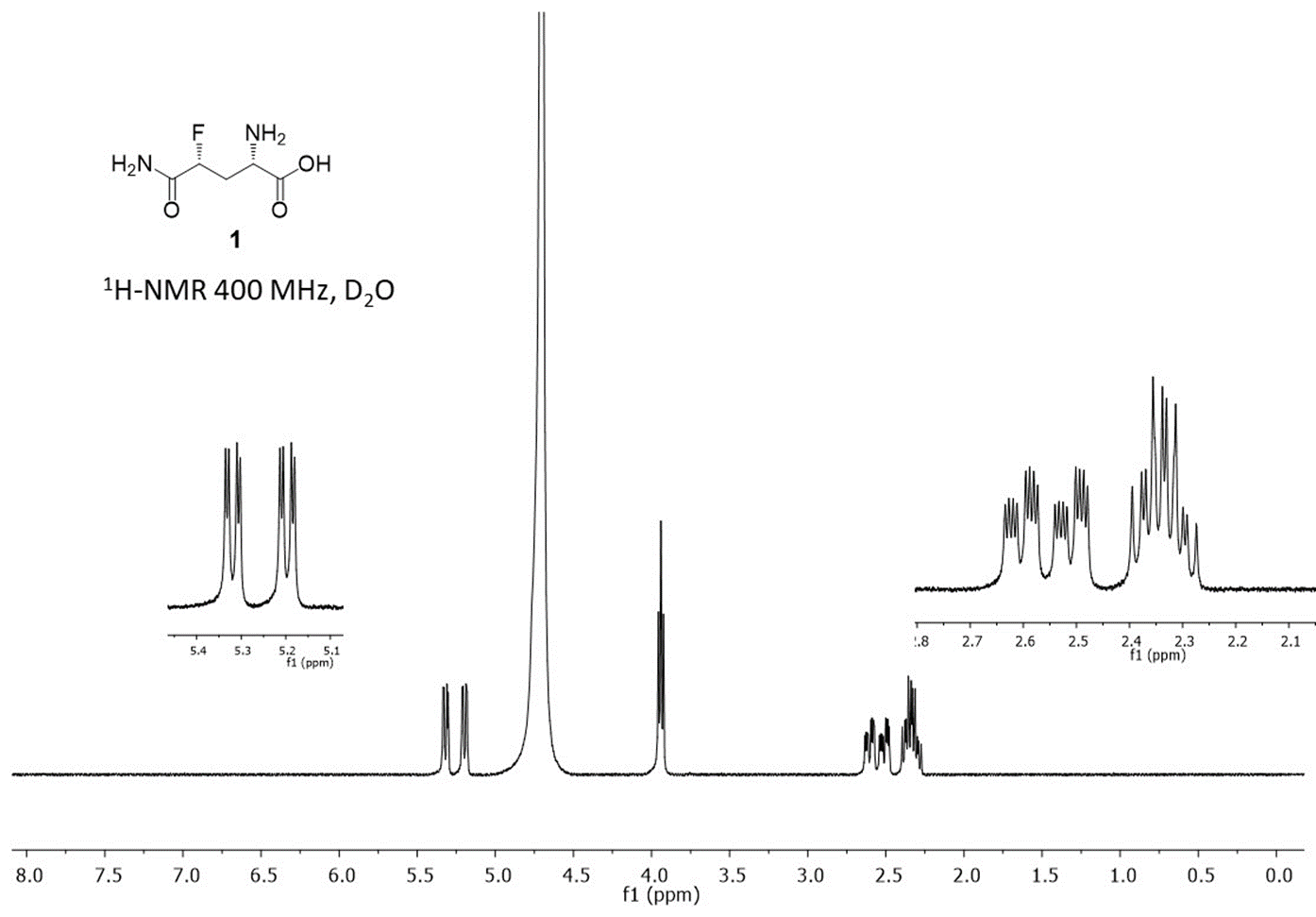
**Supplementary Figure 3:** ^19^F NMR spectrum (376 MHz, D_2_O) of compound 4-FGln **1**.


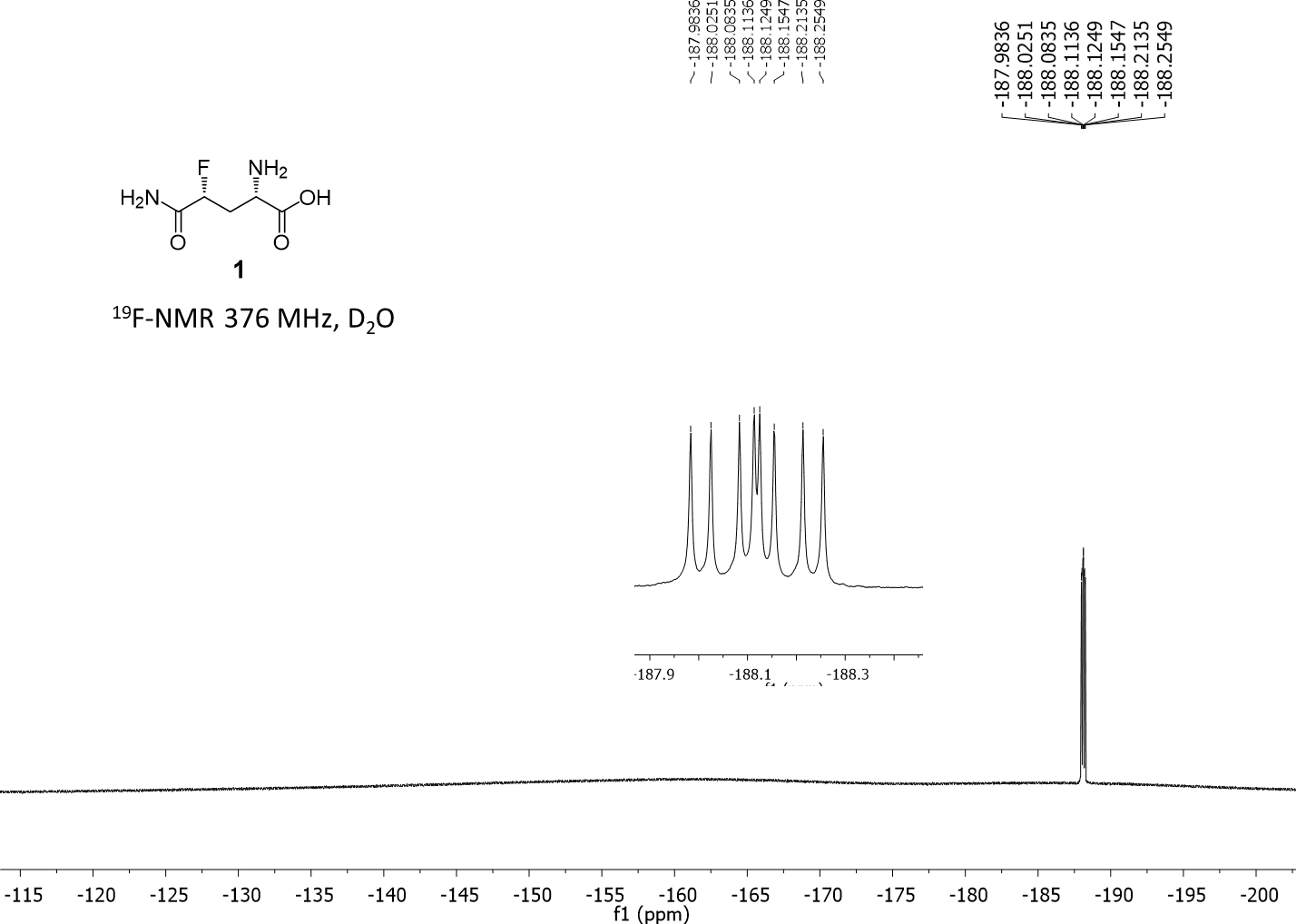


**Supplementary Figure 4:** ^1^H NMR spectrum (400 MHz, CD_3_OD) of compound **12**.


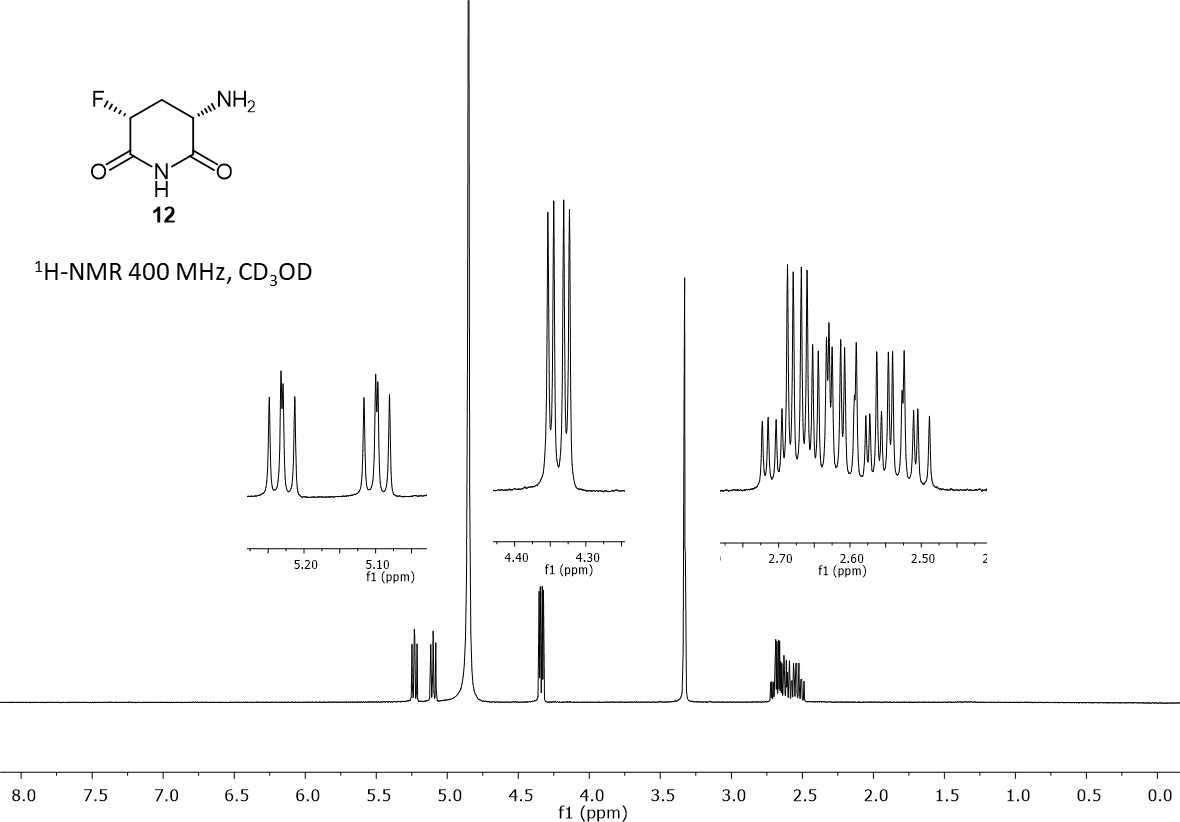


**Supplementary Figure 5:** ^19^F NMR spectrum (376 MHz, CD_3_OD) of compound **12**.


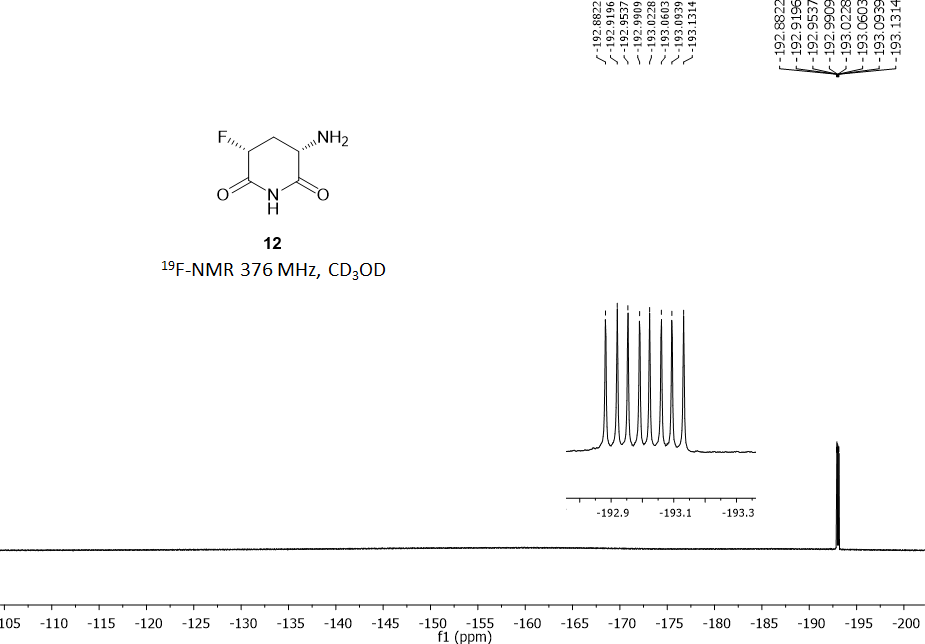

Supplement: Supplementary file 1 [file DataSheet_1.docx]
